# Othering, Stigma, and Normalization: A Key Informant Interview Study on Ethical Issues in Alcohol Policy in Australia

**DOI:** 10.1007/s11673-025-10443-6

**Published:** 2025-08-13

**Authors:** Mary Jean Walker

**Affiliations:** La Trobe University, Bundoora, Melbourne VIC 3086, Australia

**Keywords:** Alcohol, Drinking, Policy targeting, Policy inequality, Stigma, Othering, Normalization, Moralization

## Abstract

Alcohol use causes significant harms and is extensively regulated by governments, but alcohol-related questions are under-explored in public health ethics. To inform future work in this area, I conducted interviews with key informants from alcohol research and policy work in Australia. Thematic analysis of interviews identified a range of ethical issues that can be categorized under six themes: ethical issues in alcohol research; ethical issues raised by judgements about alcohol use; ethical issues raised by alcohol policies; ethical issues in policy processes; ethical questions about policy aims; and ethical issues in how alcohol problems are defined. The study provides direction for future research on problems that are underexplored in the ethics literature. These include problems arising from the influence of industry in alcohol research and policy, the question of how to develop research and policy without generating stigma, and social justice issues connected to alcohol-related stereotypes and judgements.

**Supplementary Information:**

The online version contains supplementary material available at 10.1007/s11673-025-10443-6.

## Introduction

Alcohol has an ambiguous place in Australian culture. In the 2019 household survey, 76.6 per cent of Australians over fourteen reported recent consumption (Australian Institute of Health and Welfare [AIHW] 2020), and in a 2020 poll 45 per cent of respondents who drank said they drink to get drunk (Foundation for Alcohol Research and Education [FARE] 2021). Drinking plays roles in social bonding and the creation of social meanings (Sheehan and Ridge 2001; Manton et al. 2014; MacLean 2016; Murphy et al. 2017). It also significantly contributes to health, social, and economic harms. The annual social cost of alcohol use in Australia was estimated at $66.8 billion in 2017–18 (AIHW 2023) and alcohol accounts for 4.5 per cent of the total burden of disease (AIHW 2021). Alcohol causes the most harm to others of any drug (Bonomo et al. 2019).

Alcohol consumption and alcohol policies raise ethical questions. Alcohol harms occur via three main mechanisms: addiction, intoxication (acute harms such as violence), and alcohol toxicity (chronic health harms) (Babor et al. 2010, 14). Questions related to addiction have been discussed in some detail by ethicists, while other harms have been relatively neglected. It is likely that far more harms accrue from intoxication and toxicity than from addiction. While it is difficult to quantify harms, and the three mechanisms interact, only around ten per cent of heavy drinkers are estimated to be dependent (Esser et al. 2014), indicating that significant numbers of people drink in potentially harmful ways without being addicted. Harms can follow from intoxication and toxicity experienced by the ninety per cent of heavy drinkers who are not dependent, and less frequent drinkers.

A search of PhilPapers identified only fifty-five articles discussing alcohol as a public health issue, twelve of which are brief replies to other articles on the list (Table 1). These discussions are valuable but discuss a limited number of issues, with a focus on FASD (fifteen articles/replies), ethical justification of particular alcohol policies (eighteen articles/replies), liver transplant allocation (five articles), and public health research ethics (four articles/replies). In the grey literature, the Nuffield Council on Bioethics’ pivotal report *Public Health: Ethical Issues* devotes a chapter to alcohol (2007). In contrast, Philpapers lists 1342 entries for “addiction” and 115 for “alcoholism.”

**Table 1:** Existing work on alcohol and public health ethics

	Author and Date	Title	Focus	Claim/Argument
**1**	Albertsen (2016)	Drinking in the last chance saloon: Luck egalitarianism, alcohol consumption, and the organ transplant waiting list	Liver transplant allocation decisions	Use of luck egalitarianism in just allocation of donor livers
**2**	Arkell and Lee (2023)	Using meconium to establish prenatal alcohol exposure in the UK: Ethical, legal and social considerations	FASD	Screening pregnant women for alcohol use using meconium is not justified
**3**	Banja (2014)	Alcohol and drug testing of health professionals following preventable adverse events: A bad idea	Policy for clinical ethics	Use of practices of “speaking up” as preferable to post-incident alcohol/drug tests of health professionals
**4**	Pham et al. (2014)	*Postincident alcohol and drug testing*	*Response to Banja*	
**5**	Trafimow (2014)	*On speaking up and alcohol and drug testing for health care professionals*	*Response to Banja*	
**6**	Batra et al. (2023)	The dual role dilemma of liver transplantation health care professionals	Liver transplant allocation decisions	Interview study investigating healthcare providers’ experience of caring for patients while having to monitor alcohol abstinence for transplant eligibility
**7**	Beauchamp (1976)	Exploring new ethics for public health: Developing a fair alcohol policy	Framing of alcohol problems	Against understanding alcohol problems as the result of a failure of a minority of people to control their drinking
**8**	Bell et al. (2016)	It’s a shame! Stigma against fetal alcohol spectrum disorder: Examining the ethical implications for public health practices and policies	FASD	Review of literature on stigma and FASD and ethical analysis
**9**	Bennett and Bowden (2022)	Can routine screening for alcohol consumption in pregnancy be ethically and legally justified?	FASD	Against proposed UK policy of screening pregnant women for alcohol use
**10**	Bramstedt and Jabbour (2006)	When alcohol abstinence criteria create ethical dilemmas for the liver transplant team	Liver transplant allocation decisions	The “six-month rule” (USA) can complicate rather than aid clinical decisions and is ethically problematic
**11**	Brooks (2015)	Alcohol and controlling risks through nudges	Ethical justification of alcohol nudges	Nudges to prevent harms are warranted
**12**	Bruckner (2018)	Gun control and alcohol policy	Ethical justification of alcohol regulations	Arguments for gun control in USA imply justification for stronger alcohol controls
**13**	Capron (1992)	At law: Fetal alcohol and felony	FASD	Report on law around FASD and fetal exposure in USA
**14**	Charles (2011)	Obstetricians and violence against women	FASD	Obstetricians’ advice to abstain from alcohol is similar to methods of coercive control
***15***	Partridge et al. (2011)	*In the face of uncertainty about the risks of low-level drinking, abstinence is prudent, not misogynistic, advice*	*Reply to *Charles 2011	
**16**	De Bruin (2014)	Alcohol in the media and young people: What do we need for liberal policy-making?	Alcohol advertising and young people	Evidence surrounding effects of alcohol advertising on young people is too weak to justify regulation, but could be justified on grounds of effects on autonomy
**17**	De Ville and Kopelman (1999)	Fetal protection in Wisconsin’s revised child abuse law: Right goal, wrong remedy	FASD	Evaluates fetal protection laws
**18**	Dobson and Hawkins (2016)	The idea of freedom in the policy debate on the minimum unit pricing of alcohol	Ethical justification of alcohol regulations	Identifies sense of freedom people were concerned with in interviews about minimum pricing as non-interference
**19**	Drezga-Kleiminger et al. (2023)	Should AI allocate livers for transplant? Public attitudes and ethical considerations	Liver transplant allocation decisions	Reports results of a survey of UK laypersons’ views of using AI to allocate donor livers
**20**	Gavaghan (2009)	“You can’t handle the truth”; medical paternalism and prenatal alcohol use	FASD	UK policies of recommending total abstinence are ethically problematic
**21**	Grill and Fahlquist (2012)	Responsibility, paternalism and alcohol interlocks	Ethical justification of mandating alcohol interlocks	Against two ethical objections to mandating alcohol interlocks in all cars
**22**	Helgesson et al. (2018)	Ethical aspects of diagnosis and interventions for children with fetal alcohol spectrum disorder (FASD) and their families	FASD	Positive and negative impacts of FASD diagnosis
**23**	Hu and Primc (2023)	Should responsibility be used as a tiebreaker in allocation of deceased donor organs for patients suffering from alcohol-related end-stage liver disease?	Liver transplant allocation decisions	Argues responsibility can be used as a tie-breaker in a limited set of cases of transplant decisions
**24**	Husak D (2011)	Is drunk driving a serious offence?	Ethical justification of drunk driving laws	Drunk drivers should not be imprisoned
**25**	John (2018)	Should we punish responsible drinkers? Prevention, paternalism and categorization in public health	Ethical justification of alcohol regulations	The claim that we shouldn’t punish responsible drinkers should not be used in policy
**26**	Jones (2011)	Drunken role models: Rescuing our sporting exemplars	Alcohol and sport	Elite sportspeople have an obligation to be good role models, including in drinking
**27**	Jonsson et al. (eds) (Jonsson, Clarren, and Binnie 2018)	Ethical and legal perspectives in fetal alcohol spectrum disorders: Foundational issues	FASD	Primarily law and psychology but includes 3 chapters containing examination of ethical issues related to FASD, inequity, and justice
**28**	Louise et al. (2015)	Mandatory cancer risk warnings on alcoholic beverages: What are the ethical issues	Ethical justification of cancer warning labels on alcohol products	Examines range of ethical issues related to mandatory warning labels, including autonomy of drinking decisions
***29***	Bell et al. (2015)	*Caution! Warning labels about alcohol and pregnancy: Unintended consequences and questionable effectiveness*	*Reply to *Louise et al. 2015	
***30***	Brewer and Himes (2015)	*Weighing the ethical considerations of autonomy and efficacy with respect to mandatory warning labels*	*Reply to *Louise et al. 2015	
***31***	Dawson and Mackay (2015)	*Alcohol, liberty, and societal change: What should we do about our drinking problem?*	*Reply to *Louise et al. 2015	
***32***	Plunk and Will (2015)	*How should we ethically justify alcohol warning labels? Thinking more broadly about risk, benefit, and efficacy*	*Reply to *Louise et al. 2015	
***33***	Thompson (2015)	*Evaluating public health effectiveness of alcohol label warnings*	*Reply to *Louise et al. 2015	
***34***	VanderWalde (2015)	*Cancer warnings on alcohol: Is cancer the issue and are labels the solution?*	*Reply to *Louise et al. 2015	
**35**	McCambridge et al. (2013)	The use of deception in public health behavioral intervention trials: A case study of three online alcohol trials	Public health research ethics	Discusses the reasoning for using deception in public health trials
***36***	Hendershot et al. (2013)	*Deception in human experimental and public health research on alcohol problems*	*Reply to McCambridge et al*	
***37***	McCambridge et al. (2014)	*Deception in research is morally problematic … and so too is not using it morally: reply to open peer commentaries on “the use of deception in public health behavioral intervention trials: a case study of three online alcohol trials”*	*Reply to Hendershot et al*	
**38**	Meurk et al. (2014)	A bio-social and ethical framework for understanding fetal alcohol spectrum disorders	FASD	Issues of definition and diagnosis of FASD, research needed and ethical constraints
**39**	Mills (2023)	Protecting the future child: Foetal alcohol spectrum disorder, easy rescue and the regulation of maternal behaviour	FASD	Social contexts of inequality need to be considered in debates about gestational harm to future children
**40**	Momeyer R (2000)	Heavy drinking on campus and university paternalism	Ethical justification of alcohol regulations	A policy in US university of notifying students’ parents of underage drinking is justifiable soft paternalism
**41**	Neighbours et al. (2012)	What should we do when participants report dangerous drinking? The impact of personalized letters versus general pamphlets as a function of sex and controlled orientation	Research ethics	Examines possible responses when subjects in research trials report dangerous drinking
**42**	Perry (1985)	Some ethical concerns with the minimum drinking age law	Ethical justification of alcohol regulations	Discusses several ethical concerns
**43**	Peterman and Desbiens (2005)	Should physicians be allowed to use alcohol while on call?	Policy for clinical ethics	Current guidelines against practicing medicine in USA imply that doctors should not drink while on call
**44**	Piazza-Gardner and Barry (2013)	A drink best not served: Conflicts of interests when the alcohol industry seeks to inform public health practice and policy	Industry influence on public health policy/practices	Industry involvement in policy is at odds with public health goals
**45**	Pietro et al. (2016)	Ethical challenges in contemporary FASD research and practice	FASD	Gives a framework for ethically approaching FASD research and treatment
**46**	Pratten (2007)	Reporting on responsible drinking: A study of the major UK pub-owning companies	Corporate social responsibility	Largely empirical but describes the “ethical context” of actions of pub-owning companies in UK
**47**	Price and Miskelly (2015)	Why ask why? Logical fallacies in the diagnosis of fetal alcohol spectrum disorder	FASD	Ethical issues with the attribution of causation in diagnosing FASD
**48**	Shaw et al. (2012)	Reducing the harmful effects of alcohol misuse: The ethics of sobriety testing in criminal justice	Alcohol and crime	Benefits of offender sobriety testing programme in Scotland outweigh any harms
**49**	Steinbock (1985)	Drunk driving	Ethical justification of drunk driving laws	A class of drunk drivers does commit murder
**50**	Stuart (1989)	Deterrence, desert, and drunk driving	Ethical justification of drunk driving laws	Very severe penalties for harms from drunk driving are disproportionate punishments
**51**	Swartwood (2017)	Drinking, texting, and moral arguments from analogy	Drink driving	Main focus is texting while driving; uses analogy to drunk driving to show why it is problematic
**52**	Walker (2010)	Why we should not set a minimum price per unit of alcohol	Ethical justification of alcohol regulations	Minimum pricing disadvantages the worst-off and is poorly targeted
**53**	Wilkinson et al. (2016)	Protecting children from in-utero harm	FASD	A case for intervening to prevent harm to a future child

Search strategy: Philpapers was searched in December 2023 with the query: (with at least one of the words) “alcohol drinking drunk intoxicate FASD”. A manual search of 1345 abstracts resulted in 53 entries (55 articles as #24 contains 3 relevant papers). Inclusion criteria: contains some discussion of alcohol as a public health ethics issue. Exclusion criteria: sole focus on addiction; non-ethics papers; book reviews; papers using FASD as a foil in abortion debate; papers giving empirical legal descriptions only; unpublished works; papers not in English; introductions overviewing other papers

I suggest the focus on addiction indicates under-exploration of important ethical questions related to alcohol: there seems a mismatch between where ethicists’ attention has been directed and where alcohol-related harms lie. If we examine alcohol-related harm in a broader way, questions in the remit of public health ethics will arise. Public health ethics is a developing field focused on ethical questions related to public health. Public health develops and examines practices and policies that aim to promote and protect the health of populations, inviting attention to individual, environmental, and social determinants of health as well as immediate causes of ill health. Public health thus directs attention to prevention as well as cure, and to the ways health-related goods are distributed within populations. In line with this, public health ethics often focuses on issues of justice, such as the distribution of health in a population and of healthcare, and when it is legitimate for governments to impose coercive policies for the sake of population health (Faden and Bernstein 2020).

Many forms of alcohol-related harm and policies to reduce them fall squarely in this remit. Alcohol-related harm is unequally distributed across populations and has social justice dimensions. For instance, the same level of drinking by people in lower and higher socioeconomic status (SES) groups is known to result in higher levels of harm for those with less resources (known as the “alcohol harm paradox”) (Probst et al. 2020). Some alcohol-related harms—and policy issues—are experienced differently by people in different social categories. For example, harms like alcohol-related sexual assault are gendered, and Australia has a history of paternalistic alcohol policies directed at Aboriginal and Torres Strait Islander people.

This study aimed to inform future public health ethics research on alcohol, by identifying ethical issues related to alcohol policy in Australia. A second aim was to understand how ethical issues are discussed, framed, and understood by people working in alcohol policy.

## Methods

A key informant interview design, focused on recruiting people with expertise on Australian alcohol policy, was adopted to meet the above aims. A qualitative study was adopted as fitting the exploratory nature of the research. The study was conducted using Braun and Clarke’s method of reflexive thematic analysis (2006, 2019), which is designed to be relatively neutral to theoretical commitments. As I sought considered judgements, I decided to consult key informants who were professionals working in alcohol policy or alcohol policy research. The study aimed to include perspectives from different locations in Australia and (for researchers) disciplinary backgrounds.

Policy workers and researchers who had significant expertise related to alcohol from across Australia were invited to participate. Informants were approached by email, directly in the case of researchers, and via organization in the case of policy workers. Sixty-two invitations were sent (seventeen to organizations). Policy organizations were relevant state and federal government departments and NGOs (peak bodies and advocacy organizations). I decided not to seek informants from alcohol industry bodies because there is significant distrust of such bodies, and research that engages with them, among policy workers and researchers, such that their inclusion could discourage other potential informants from participating.

Informants were primarily researchers (thirteen informants) rather than policy workers (four informants). Seven informants had professional experience in both areas, and among researchers who had not been employed in policy positions, some had been involved in policy at the level of advocacy or consulting for government or national/international health bodies. Informants’ sociodemographic data was not sought because doing so would pose problems for informant anonymity, given the small pool of potential informants. As the aim of the study was to draw on expert insights to identify ethical issues rather than to uncover an ethical perspective representative of a group, this is not problematic. Ethics approval was obtained from La Trobe University’s Human Research Ethics Committee (HEC21242).

Seventeen semi-structured qualitative interviews were undertaken between July and November 2021. Interviews were conducted and recorded over Zoom (while some areas were in Covid-19 lockdowns) and lasted between forty-three and ninety minutes. Informants chose their physical interview location, and none were accompanied by others. Participants received an information and consent form, which included information on the study aims and my research background, before the interview and were invited to ask questions.

Interviews were guided by prompts asking informants for their view of whether and how ethics and values were discussed in or informed their work, and what they thought the most important ethical issues related to alcohol in Australia were (see appendix 1). Extension questions asked for examples or elaboration of claims informants made (e.g., about particular policies being problematic), clarification of what made something “count” as an ethical issue in the informants’ view, and clarification of how the informant understood any value-related terms they used (e.g. “rights,” “responsibility,” “equity”/“equality,” “justice,” “blame”).

Recordings were transcribed by a secure service and imported to NVivo. Transcripts were not returned to interviewees for correction to avoid adding to participant burden but were extensively checked against recordings.

Themes were generated inductively. An initial 628 codes were recorded against transcripts and then iteratively reviewed and organized into themes. This resulted in six themes that contribute to answering the research questions. The themes are similar to what Braun and Clarke call “domain summaries” (summaries of different ideas relating to a particular topic) but were generated by the analysis and are not merely summaries of responses to interview questions (Braun and Clarke 2019). Given the purpose of the study, to identify ethical issues, domain summaries capturing a range of issues and different perspectives on them within the sample are appropriate.

In terms of reflexivity, I am an experienced ethics researcher and teacher with specialization in bioethics and experience in qualitative analyses and have worked in alcohol and drug policy. I avoided introducing ethical terms or ideas and focused on exploring those introduced by each informant. I also tried to remain aware during interviews and analysis that as an ethics teacher and researcher I may tend to conceptualize ethical problems in ways reflecting that intellectual background. I had no relationship with interviewees.

The below descriptions note roughly what proportion of informants raised particular issues (“several”: 2–4, “some”: 5–8, “many”: 9–2, “most”: >13). However, selection of themes was guided by significance in relation to the research questions, not by prevalence of an idea in the sample (Braun and Clarke 2006).

## Results

Themes, sub-themes and issues are summarised in Table 2.

**Table 2:** Themes, sub-themes, and identified issues

Theme	Sub-Themes	Issues Identified
Ethical issues in alcohol research	N/A	• Industry funding and its management • Impacts of stigma in research ethics process • Generation of stigma via research • Research representativeness • Embedded assumptions in datapoints/outcome measures
Ethical issues relating to judgements of alcohol use	N/A	• Generation of stigma • Social coercion to drink • Unfair/inconsistent judgements of acceptability of drinking • Potentially negative changes contributing to changing drinking habits of young people
Ethical issues with current policy	Harmful policies	• Particular policies causing harm (e.g., stigma, distress/trauma, debt, criminalization) • Lack of anticipation of policy harms among policymakers
	Inequitable policies	• Racially discriminatory policies • Policies discriminatory in application • Distinguishing acceptable/unacceptable inequalities in policy
	Policies that infringe rights	• Infringements of privacy from public health surveillance • Uses and biases of AI predictive modelling in public health • Mandatory treatment
	Unjustified policies	• Policies not justified by evidence • Policies not most beneficial option • Policies not justified by a warranted aim
	Issues in need of (better) policy	• Lack of sufficient advertising regulation (especially for children) • Difficulty regulating online marketing • Marketing targeting the vulnerable via social media • Home delivery of alcohol
Ethical problems in policy processes	Industry influence	• Vested interests skew public health advice and decisions
	Policy inconsistencies	• Evidence/policy gap • Inconsistency of alcohol and illicit drug policy
	Cultural ideas and political realities	• Political and cultural factors result in preference for less effective policies • Moral judgement of alcohol use/problems and related social stereotypes still drive policy choices
What should policy aim to do? Normative claims and questions	Less coercive policies	• Information provision/education as obligations of governments • Underutilization of harm- and demand-reduction
	Alcohol controls	• When is paternalism justified in alcohol policy? • Is alcohol use always harmful to others in the sense required to justify controls? • When if ever are alcohol-related choices free? • What senses of freedom are negated or supported by different options for alcohol control?
Ethical issues in defining alcohol problems	Othering and normalization	• Othering individualizes alcohol-related problems, mis-characterizing their causes by omitting systemic and structural determinants • Othering is associated with stigmatization and blame for problems • Normalization of alcohol use/intoxication contributes to unacknowledged or accepted harms • Othering and normalization reinforce each other but attempts to challenge them can have other negative effects
	Limitations of public health approaches	• A need to recognize non-health values • Polarization of debate due in part to industry actions

### Ethical Issues in Alcohol Research

Some informants raised concerns about research funded by the alcohol industry, questioning its objectivity and whether companies use it to present themselves as ethical. Several researchers questioned whether journal requirements for disclosure of industry funding are typically adequate, whether disclosures are always made, and whether disclosure is effective in preventing issues related to conflicts of interest.

Many researchers noted the need to approach alcohol research in ways that would not stigmatize any groups. Stigma could also generate research ethics issues. For instance, research with Alcoholics Anonymous participants could be complicated by HREC requirements for names on consent forms. Stigma might discourage people from participating in studies, which can affect understanding of the prevalence of a problem or undermine study representativeness. Several said that devaluing of research participants who are “dependent”^1^ sometimes leads to a view that they need not be compensated for their time or are likely to be unreliable, including among HREC members.

Several researchers raised problems with the representativeness of alcohol research. Alcohol treatment remains based on historical research done with men and there is a lack of data on approaches effective with women. Alcohol use research often recruits from treatment centres, which may not be a representative population.

All informants were reflective on ways values can direct and impact on research and its use. Several researchers noted ways research can be partly driven by contingencies of what data is available or what form it is in. For instance, government datasets from health and welfare services were thought to embed assumptions (about people or substances) in the way datapoints are classified:

… often we put people into boxes or codes that are defined by someone outside of them … I think we need to think about those classifications regularly and review them and think about how we use them. *[Informant B]*

Involving participants in study design was raised by several researchers as a means to examine value assumptions and generate alternative approaches to classifications.

### Ethical Issues Relating to Judgements About Alcohol Use

While several informants reflected on the ethical acceptability of drinking alcohol, most said we should not judge people’s drinking to avoid stigmatization. The difficulty of how to address alcohol-related harm while avoiding stigmatization was discussed as a major ethical challenge.

Several informants suggested it is an ethical problem that people can be socially pressured to drink, noting that drinking and intoxication are part of Australian culture. A range of social meanings of alcohol use were noted: brands can be markers of status or associated with a sport or profession, and drinking can signal a shift in role, solidarity with others, or personal freedom. Thus, drinking can be used to construct identities and generate cultural meanings, and this can affect people’s drinking choices. The alcohol industry was said to encourage these associations. Further, cultural beliefs around how people who are intoxicated will behave (e.g., the belief that alcohol leads to aggression) were said to play a role in generating alcohol harms.

The culture that accepts intoxication as being okay makes it very difficult for people who are intoxicated to actually remain responsible, to remain able to make decisions. *[Informant G]*

However, several noted there are also social norms against particular drinking practices and contexts. One informant suggested this creates an ethically problematic situation in which people are encouraged to drink, then judged for it.

… particularly young people really know that they live in a world where they’re very much encouraged to drink. But the point at which, when they do that, they are judged, varies according to a whole lot of factors that sometimes are out of their control. *[Informant F]*

Further, many informants thought that, since the ways drinking is judged can differ depending on where, with whom, and when drinking occurs, or features of the drinker (gender, perceived race/ethnicity, etc.), people’s judgements about drinking are contingent on arbitrary social norms. As a result, judgements of when alcohol use is problematic do not align with when drinking is in fact harmful.

… the NT [Northern Territory] has insane rates of drinking, with the second highest rate of drinking in the world across the population … that's very much seen as an Aboriginal problem … which is absolute crap … the non-Aboriginal population up here drink like fishes. … if you drink behind closed doors in a private property or in a bar, no problem at all, you can be the biggest alcoholic and abusive piece of crap in the world, and no one will blink at it. Someone sitting in a park drinking, completely rounded on by the general public and seen as the source of all social problems. *[Informant M]*

Several informants commented that women are stigmatized for drinking in ways that men are not: “in terms of messiness, and impurity … and particularly relating to mothering” *[Informant O].* One researcher noted that alcohol-related judgements often reflect other social inequalities, so that the drinking of people in marginalized groups is judged more harshly.

Some informants noted recent changes in drinking patterns, with younger people drinking less. While research on this trend is still underway, it was connected to increased knowledge of health risks, increased social media use, changing modes of identity construction, increased risk aversion, less happiness among young people, and increased drinking stigma.

### Ethical Issues With Current Policies

Informants raised ethical concerns about specific policies where they cause harm, are inequitable, infringe rights, or are not well justified. Concerns were also raised about some issues in need of better policy.

#### Policies Causing Harm

Many informants raised concerns with policies that they argued generated stigma. Examples included the Northern Territory “Intervention” and Stronger Futures legislation, which imposed alcohol restrictions on people in prescribed areas in the Northern Territory from 2007–2022 (CCHRL 2020); cashless welfare cards, which are partly motivated by reducing spending on alcohol (Services Australia 2024); and anti–public-drinking laws.^2^

… knowing every time you go to the shops, you have to hand over the Basics [cashless welfare] card and there's judgement. *[Informant D]*

Stigma was said to contribute to other harms, by increasing risk factors for higher and more harmful consumption.

The Intervention/Stronger Futures was also said to have caused trauma and distress, people leaving children unsupervised to travel elsewhere to drink and harms to communities from removing community-owned alcohol measures. Cashless welfare sometimes caused people to go into debt. Restrictions on alcohol use in remote working areas could cause shifts to using more dangerous substances. The interaction of different policies and bureaucratic processes could also lead to harms, e.g., people not accessing dependence treatment from fear it would generate child protection interventions.

Some of these harms were said to arise because policy tends to be made by more powerful people whose experience of alcohol-related harm is likely to be lower (even at the same levels of consumption), implying they are less able to anticipate negative effects of policies. Several informants suggested that community involvement in policy development would help to better anticipate harms.

#### Inequitable Policies

Policies affect different population groups differently, intentially or not, raising issues of inequity and discrimination. Many informants considered the Intervention/Stronger Futures to be racially discriminatory. Cashless welfare policies were also suggested by one informant to be racially discriminatory, as they have been trialled in areas with higher levels of Indigenous residents.

The powerful are always willing to do things to the less powerful that they're not willing to do themselves. *[Informant H]*

Policies that are in-principle applicable to everyone also affect groups differently. Inequality can occur when a policy involves the discretion of police or other actors and so may be applied in ways reflecting social biases. Examples included area-targeted policing (Birks et al. 2023), face-scanning/blacklisting of customers in nightlife venues (QLD) (LECC 2023; Miller et al. 2023a, b), police auxiliaries in bottle shops (NT) (Office of the Chief Minister of the Northern Territory 2018), Banned Drinker Registers (BDRs) (NT, WA) (DLGSCI 2024; Northern Territory Government 2024), and child removal linked to stereotypes about FASD prevalence in different groups. One policy worker proposed that Aboriginal people are more likely than non-Aboriginal people to be referred to alcohol treatment, rather than mental health treatment, for the same presentations.

[Under the NT police auxiliary policy] before you go and buy the alcohol, in theory, everyone gets screened before you walk into a bottle shop. But Aboriginal people are more screened. *[Informant D]*

Anti-public drinking measures were seen by several informants as de facto racially targeted since Aboriginal people are more likely to drink outside. One informant suggested these may also be unequally applied: while enforced against Aboriginal people, police might “turn a blind eye towards … the champagne at the picnics” of non-Aboriginal people *[Informant O].*

One researcher spoke of the way Aboriginal people and communities have been, over time, targeted by numerous intersecting policies, which can be inconsistent and unclear, and the cumulative effect of this on resilience.

A policy’s being inequitable often implied it was ethically questionable, but there were exceptions. Many informants thought that alcohol measures in Aboriginal communities are ethically acceptable when they are community-owned (developed by and advocated for by the community). Several thought that increasing alcohol prices, which is economically inequitable, is justified because price rises affect people from lower SES groups more and these groups suffer more alcohol-related harm. One researcher noted that particular policies may also need more complex analyses recognizing intersectional interests; e.g., a price increase unpopular among lower-SES men might benefit lower-SES women.

#### Policies That Infringe Rights

Some informants noted issues with public health surveillance infringing privacy. These included use of government databases to implement BDRs; tracking individual purchases (as part of the BDR; NT) (Northern Territory Government 2024); face-scanning in nightlife districts (Miller et al. 2023a, b); and uses of linked data from welfare, AOD, and other government services (AIHW 2024). With regard to the latter, there was concern about using AI analyses of these databases for predictive modelling of who is likely to run into problems, particularly among disadvantaged groups.

Several informants raised concerns about mandatory treatment laws in some states (Vuong et al. 2019).

#### Unjustified Policies

Several informants thought there were policies that were not sufficiently justified. Examples included anti-public drinking measures (as public drinking does not in itself cause harm) and BDRs (some of which prevent someone buying alcohol but do not provide support).

#### Issues in Need of (Better) Policy

Many informants argued for more stringent regulation of alcohol marketing, criticizing the current system of industry self-regulation (ABAC Scheme Limited n.d.). Some were particularly concerned about advertising targeting children and young people, currently enabled by a regulatory loophole that allows alcohol advertising during sporting events.

Many noted concerns with difficulties regulating online marketing. One issue here was that social media algorithms target advertising toward young people and high-volume consumers. Such marketing was said to prey on vulnerable people “by design” *[Informant P]*.

Some informants noted alcohol home delivery as an emerging issue in need of better regulation.

### Ethical Problems in Policy Processes

Informants raised ethical concerns about policy processes related to industry influence, inconsistencies, and the impact of cultural ideas and political realities.

#### Industry Influence

Most informants saw the influence of alcohol industry bodies on policy as the major ethical issue related to alcohol in Australia. The alcohol industry was said to influence policy at every level via lobbying, submissions to inquiries, and presence on advisory bodies. Industry was also said to influence policy by interfering in public debate, funding research to skew the evidence base, promoting biased evidence, and undermining respect for peer-reviewed evidence amongst policymakers. Industry-run measures (e.g. campaigns around drinking while pregnant) were seen as disingenuously twisting health messaging.

Several informants with policy experience noted that it is appropriate for policymakers to consider impacts of policies on producers and sellers of alcohol and consult with industry on their business implications. However, it was thought industry influence goes far beyond this, influencing health policies on which they lack expertise, and with which they have a conflict of interest. Several informants presented their research or policy work as a “war” against the alcohol industry.

The alcohol industry is … promoting to young people and seeking to oppose measures to reduce alcohol harm … I think “evil” is about the right word to use for it. It’s not even an emotive term. It’s just factual.” *[Informant A]*

Several pointed out that current practices are inconsistent with the exclusion of tobacco industry representatives from public health consultations under the UN Framework Convention on Tobacco Control, to which Australia is a signatory. Most concerns about industry influence focused on its health implications, but one researcher spoke of the commercialization of cultural spaces by alcohol companies as a harm to cultural life.

#### Policy Inconsistencies

Many informants commented on an “evidence/policy gap”: alcohol policies adopted are often at odds with evidence about alcohol harms and how to reduce them. The main cause of this gap was said to be industry influence.

We know the dangers of alcohol use. We have a fairly good idea from research, what kinds of policy can influence alcohol use. … Policy isn’t keeping up with that. Policy is to let people drink as much as they want, pretty much. *[Informant K]*

Several informants considered it problematic that alcohol policy is inconsistent with illicit drug policy.

#### Cultural Ideas and Political Realities

Some informants noted that the most effective policies to reduce alcohol-related harms—price and availability controls—are unpopular and politically difficult to introduce. Policies to intervene in the social determinants of consumption or harms (e.g., preventing trauma) are long-term, difficult, and expensive and, because they work via prevention, difficult to substantiate as effective. Thus less-effective group-targeted policies tend to be selected. Punitive policies are more likely to be popular than those providing support to stigmatized populations and can be politically expedient.

Moral judgements of drinking were thought to play a role in encouraging targeted policies, as people judge the drinking of less powerful groups more harshly. Moral views were also thought to underlie the inconsistencies between alcohol policy and illicit drug policy. The moral model of addiction (regarding addiction as a moral fault) was regarded as outdated but still driving policy and popular opinion.

### What Should Alcohol Policy Aim to Do?

Many informants saw implementing less coercive alcohol policies as an obligation of governments, and many argued for more coercive policies including tighter controls.

#### Less Coercive Policies

Many informants argued that governments have obligations to enable individuals to make informed choices about alcohol by providing information and education and restricting alcohol marketing.

Many also argued for a need for more demand- and harm-reduction policies (in contrast to supply reduction).^3^ Several stated that reducing consumption should not be regarded as a policy aim in itself, but merely instrumental to reducing harm. Several favoured demand reduction as a prevention strategy, arguing policies should aim to support people’s capacities and opportunities to be healthy and live a good life, such as measures to promote employment and recreation.

You don’t just take a soporific out; you actually create a more meaningful life. *[Informant N]*

#### Alcohol Controls

Some thought that less coercive policies are ethically preferable as they do not interfere with individuals’ choices. However, many informants also thought more coercive controls on alcohol availability are justified. While there was recognition of the need to consider the economic impacts of alcohol controls, several stated that health considerations should be prioritized.

We’re really mostly focused on trying to reduce the amount … we’re not trying to shut down their [venues’] businesses, but it’s not right, I don’t think it’s right that their business model requires excessive drinking. *[Informant C]*

The main justification of more restrictions to which informants appealed was harms to people other than the drinker. Several informants appealed to the notion that alcohol is “no ordinary commodity” (an approach from Babor et al. (2010)), i.e., different from other products due to its psychoactive properties.

It belongs more in the category of pistols, than it does in the category of peas and milk. *[Informant H]*

Many stated that consumption that does not harm others should not be restricted, though there were several informants who endorsed paternalist restrictions to reduce harm to drinkers. A preference for freedom limited by harm to others is perhaps not unexpected in a liberal democracy. However, some informants offered three further claims to justify having more coercive policies.

First, several informants argued that alcohol use always harms others. Even if only the drinker experiences direct harms from their drinking, it will have broader costs to others: minor “harms” like tactless remarks or discomfort from intoxicated people in public and diffuse costs such as increased societal healthcare spending. One researcher also suggested that drinking harms others by triggering others to drink.

Your drinking harms the person next to you because it instils an automatic response of drinking. *[Informant J]*

As another informant put it, a case where someone’s drinking never harms anyone else “either never exists, or it’s so rare as to be irrelevant” *[Informant G]*. Several other informants, however, noted hesitations about such arguments. These related to the difficulty of attributing causation for multifactorial chronic health problems to alcohol consumption, whether minor impacts on others should count as “harms,” and discomfort with the idea that individuals should be required to change their behaviour to protect others in such ways.

Second, some argued that alcohol restrictions are justified because choices to drink alcohol are not fully, or (for several researchers) at all free. This was said to be true for individuals whose freedom or rationality is limited by dependence, previous trauma, youth, or intellectual disability. Some argued that drinking choices more broadly are unfree because they are influenced by marketing and social coercion.

… personal choice is limited when we live and work and play in environments that are wallpapered with alcohol promotion … do you really have that full freedom to choose, when you’re seeing alcohol promotion everywhere you go? It’s targeted to you, and you actually can’t avoid it. You can’t turn it off. And when you can’t even get your hair cut or go to a one-year-old’s birthday party without alcohol being offered to you, is it really a choice?” *[Informant C]*

… we live in an intoxigenic environment. *[Informant I]*

One researcher argued that regarding alcohol access as an important freedom is a contingent result of historical alcohol restrictions for oppressed groups (e.g., restrictions on Aboriginal and Torres Strait Islander people’s access (Brady 2007) and restrictions on women drinking in pubs (Kirkby and Luckins 2006)). Another argued that our view of alcohol as pleasurable relies on learned associations, whereby drinking decisions are linked to subconscious psychological triggers that bypass rational decision-making. They proposed a thought experiment of a society in which socializing involves, not drinking, but regularly hitting oneself on the thumb with a hammer, arguing that in such a society,

First of all, you would associate hitting your thumb with a lot of pleasure. And then secondly, you would say, “Well, why should we not bring our hammer to the neighbour’s to hit ourselves on the thumb? That’s the tradition, and there’s always a lot of enjoyment going on.” You would like to restrict, you would like to take my hammer away from me. And I would say, “It’s good for your thumb, it doesn’t hurt, … it was so much fun with the neighbours.” *[Informant J]*

These several informants then regarded the association of alcohol with freedom, pleasure, or other benefits as, at least in part, culturally constructed and learned. This was a minority view: most informants thought that alcohol can have benefits, including pleasures:

… especially for young people who aren’t drinking heavily, good to get them outside, good to get them bonding with young people away from their screens, talking, lowering their inhibitions a bit, and being able to talk to those they’d usually be too shy to talk to … *[Informant O]*

Third, some informants argued alcohol controls are justified because they support other freedoms: freedom from harms from others’ drinking and freedom from social coercion to drink.

Alcohol is everywhere. It’s very exhausting to be constantly choosing. *[Informant F]*

One informant argued with respect to particular Indigenous communities that community-led local restrictions can support not individual freedom but a community sense of self-determination.

### Ethical Issues in Defining Alcohol Policy Problems

Informants commented on issues related to how alcohol policy “problems” come to be defined as such. Comments centred around ways problems are either defined as occurring only for particular individuals or groups (othering) or not recognized as problems (normalization). Some also commented on limitations of public health approaches to understanding and resolving alcohol-related issues.

#### Othering and Normalization

Many informants argued that alcohol problems tend to be framed as problems located in particular individuals or groups. This implies the problems are those of people defined as “other,” abnormal. Othering was aligned with a tendency to individualize alcohol-related problems—to see them as resulting from the choices of individuals—and to see people as to blame for difficulties they experience.

One way informants talked of problems being othered was through the concept of addiction.

[Our society’s narrative around alcohol is that] it’s a small proportion of people who are strange and weird and wonderful; they’ve got something genetically wrong with them, they’re bad, they’re naughty. And the rest of us, drink as much as we like, because we’re all okay, we’re normal … *[Informant N]*

Addiction individualizes problems irrespective of whether it is seen as a physiological state (the “disease model”) or a moral failing (the “moral model”). The moral model was seen as deeply problematic, but several informants argued that the disease model can also be othering. Several noted that blame for addiction can be stronger if we do not acknowledge its psychological and social (as well as biological) determinants and suggested adopting a “biopsychosocial” model. However addiction is conceptualized, seeing “the problem” of alcohol as primarily about addiction locates it in a non-normate group.

Several participants noted that what counts as addiction is socially decided. The designation of someone’s drinking as addictive can be biased by factors like their perceived race/ethnicity, gender, age, location (drinking outside), or pattern (drinking alone). That is, while “addicted people” is an othering category, it can be expanded so that any “problematic” drinking is regarded as addictive.

… we’ve all got, in our mind, this concept of addiction, which is a person who has reduced or impaired control, and is therefore the opposite of the ideal neoliberal person who manages their own life and successfully negotiates risk. So, I think we kind of can jump to that trope of addiction to explain any kind of drinking, where it doesn’t seem like it’s under control, and where people become rowdy or unruly. *[Informant F]*

Forms of non-addictive drinking (e.g., binge drinking) can also be othered. This could involve judgements of individual drinking as irresponsible or designating particular people as “vulnerable” to alcohol-related problems.

People are aware that other people might have alcohol problems or may experience harm from alcohol, but not me. I’m a responsible drinker. No matter how much people drink, they generally consider themselves a responsible drinker … everyone thinks that they’re fine. But others over there might have a problem. *[Informant C]*

Some informants suggested that othering occurs because people prefer to believe they are not vulnerable to problems or not obligated to help.

We’re all just one disaster away from being homeless really. Like you think about so many things could change in our world that some of us are lucky enough to avoid … others don’t get that luck. And I think that frightens people, so they don’t want to look at it in that context. It’s easier to say, “Well, if he’d never started taking drugs, or if you would just stop taking drugs, or stop drinking, the problem would go away.” *[Informant B]*

Othering was said to promote an inaccurate understanding of alcohol-related problems, because problems do not really reduce to those of addiction.

… in a lot of people’s minds … most of us drink moderately, reasonably, with self-control. And it’s a minority of people … who are dependent, but the rest of us are okay. Whereas in fact, the evidence tells us that … it’s not a few people … If I believe that alcohol problems only affect a small minority of people who are alcoholic, and I’m definitely not an alcoholic, then those things reinforce each other. *[Informant N]*

Instead, alcohol-related problems are connected to patterns of drinking that are widespread and normalized. Processes of othering allow us to ignore this. This contributes to and reinforces normalization of whatever drinking is *not* deemed addictive. In this way “normal” drinking is precluded from being a public health problem by definition—even though it does cause harm. Several informants argued that Australia’s drinking culture alters our understanding of what alcohol-related harms are acceptable or even renders them largely invisible.

Othering was also said to be unhelpful in distracting us from the role of social and structural features in problems. It thus invites policy responses focused on either treatment (if alcohol-related problems are regarded as disease), or more broadly, on ways to change the choices of particular individuals or groups. Thus, it invites policies targeted by population. These are less effective than population-wide regulations or interventions into the social determinants of harms. Further, othering stigmatizes individuals who are dependent, drink in problematized ways, or belong to stigmatized groups.

Othering of alcohol problems on one hand, and normalization of harmful drinking on the other were in this way suggested to be bound up with each other by some informants. While this may suggest that a broader range of alcohol-related harms should be recognized, and normalized practices questioned, one informant warned strongly against

… wholeheartedly problematizing alcohol without nuance. … alcohol can be beneficial for a lot of people, I don’t think it’s necessarily a terrible substance. I can understand that there are so many complexities … But I don’t think young people going to nightclubs and getting really drunk and having a good time is something we should stop them doing if it’s bringing them pleasure and joy and social bonding. *[Informant O]*

#### Limitations of Public Health Approaches

Several informants argued for a need to better take non-health-related values into account. For example, it was argued that even if a policy of face-scanning at nightlife venues does reduce harms, it fails to intervene in the underlying problem:

… the massive profiteering from it that’s going on in that space [nightlife], and what that does to its cultural diversity and cultural forms that are possible within it … if our answers are always going down that [surveillance] path, and those modes of control, then we’re not thinking that the cultural dimension of this very well … you need more than just that health and harm lens. You need to … have a broader, deeper account of people’s lives and the kind of world they want to live in.” *[Informant P]*

Some researchers noted that public health debates can be limited because public health professionals cannot recognize the positive elements of alcohol consumption, because such statements from them could be misused by the alcohol industry.

It’s difficult as a public health researcher to acknowledge that alcohol use results in enjoyment or it’s done for enjoyment, because there is a powerful industry and they invest billions of dollars each year to promote enjoyment, and to make us think it’s all about enjoyment. *[Informant J]*

Several stated that this can push public health professionals toward focusing on reducing drinking as a non-instrumental aim and contributes to a loss of nuance and polarization of debate.

## Discussion

The study identified numerous ethical issues related to alcohol policy where there could be fruitful application of conceptual resources developed in public health ethics. There is not space to examine all the issues here, and the aim of this study was to identify issues for future focused research. Here I therefore discuss what these findings indicate for further useful contributions from ethicists. Broadly speaking, the study indicates that alcohol policies can often raise ethical concerns by doing harm (even while preventing other harm), contributing to inequalities, or contributing to stigma and stereotyping and that policy selection is directed by an array of drivers other than improving health and well-being. This indicates the value of further work by ethicists in this area, including on how ethicists can work within policy processes to aid in developing ethically justifiable policies.

Among the issues identified by informants, only a few appear in the public health ethics literature: the justification of particular alcohol policies, especially coercive policies (twelve full articles, six replies and one grey literature chapter), the role of industry actors (two articles), alcohol advertising (one article), and issues of defining alcohol policy problems (one article). Other concerns have not yet been explored by ethicists in detail, though broader debates in public health ethics could be fruitfully applied to them.

The issue of industry influence—on research, policy, aims, and definitions of problems—stood out as a major source of ethical concerns among the informants. An empirical literature examining alcohol industry actions is beginning to be generated (e.g., Avery et al. 2016; Miller et al. 2021; Miller et al. 2023a, b) but only two ethics papers have as yet examined this issue (Pratten 2007; Piazza-Gardner 2013). Ethical investigations of the influence of corporate interests in other areas—for instance of pharmaceutical companies on medical research and practice—and related theoretical work on corruption and conflicts of interest could provide useful resources to extend this discussion (e.g., Moore 2005; Jorgensen 2013; Kaplan 2023).

The issue that stigma—and through it, harm—could be generated by public health efforts, was also present in several themes. This will be difficult to avoid insofar as the motivation to target some phenomenon in policy (or research to inform policy) will usually arise from negative evaluation of the phenomenon. A similar difficulty is encountered in thinking about medicalization: the aim of medicalizing a particular condition may be to identify targets for intervention or support, but stigmatization of the condition can result (Kukla 2014). There has been some recent work on this issue in other areas of public health ethics (Harwood et al. 2022) and in relation to addiction (Matthews et al. 2017), as well as a broader literature in ethics seeking to understand mechanisms of stigma. Further investigation of this tension in relation to alcohol policy taking a normative perspective, and examining the issues beyond addiction, would be of use. The ways that judgements of alcohol use are affected by factors like the drinker’s social identity—and that judgements are often harsher for those with marginalized identities—also indicate the need for intersectional normative analyses, not yet present in the ethics literature.

Issues of social justice appear across the different themes, with inequalities related to race, SES, and gender of particular concern. Even where efforts are made to avoid inequitable policies, it appears social biases can enter into policy development, selection, and application. This may suggest that dominant utilitarian approaches in public health will be limited in attempts to formulate ethical policy, insofar as they focus on population levels of health and harms rather than their distribution. Social justice or relational approaches to public health ethics (Baylis et al. 2008; Powers and Faden 2008) could be of use in further examining how alcohol policies could come to reinforce other social inequalities.

There is also work in public health ethics that is not focused on alcohol, but that articulates similar concerns to those voiced by informants about what counts as a “harm to others,” and whether coercive policies can support some sense of freedom (e.g., Jennings 2009; Griffiths and West 2015). While the tension between individual freedom and public health goals is one of the main focuses of the existing ethics literature, and this work is valuable, existing papers primarily focus on assessing particular policies in different countries and contexts. Systematic approaches to balancing health and freedom concerns in relation to alcohol policies might be further developed.

Further, there are reasons to broaden the scope of ethical debate relating to alcohol. The study informants provided reasons to think there are ethical questions to ask about the way that alcohol problems or harms are identified. Beauchamp (1976) argued nearly fifty years ago against understanding alcohol problems to be problems of a minority, or of individuals, but rather as broader public health issues. This advice still resonates with the othering/normalizing tension described above. Indeed, it is possible that tendencies to either “other” problems or not recognize them as problems explain ethicists’ focus on addiction, which arguably both reflects and reinforces the idea that alcohol-related problems are located in addicted individuals, while other consumption is problem-free. The focus in the existing literature on FASD, with fourteen articles identified—and although this is of course an important issue—may also indicate a preoccupation with understanding ethical issues in terms of personal responsibility among ethicists.

## Limitations

Key informant studies do not provide generalizable information (Akhter 2022). Only alcohol policy workers and researchers were included in the study and most informants saw themselves as public health professionals. Their views may tend to reflect commitments to the aims and orientation of public health, but they cannot be regarded as representative of this profession or any other group. Examination of other perspectives such as suppliers, politicians, or laypeople might reveal different issues.

While the sample included both policy workers and researchers, no government policy workers responded to invitations (though several informants did have previous government experience). Further, although informants were drawn from across Australia, alcohol-related issues differ significantly within Australia’s large jurisdictions, so issues for particular locations would not have been identified.

Key informant interviews are conversations and as such can be affected by the interviewer’s preconceived ideas or priorities, despite the interviewer’s intention and best efforts, given the difficulty of conducting a truly non-directive conversation (Akhter 2022). In ethical discussion it is usual to use “parroting” and paraphrasing (repeating one’s understanding of what someone means back to them) to check meaning and invite elaboration. It is possible this introduced terms or ideas into discussions that informants would not have chosen, though effort was made to only use more explicitly value-related terms in ways introduced by the informants.

## Conclusion

This study identified a number of alcohol policy issues that have an ethical dimension, which could benefit from applying resources developed in public health ethics. Ethicists are well placed to help untangle differing value commitments, and there are reasons to think it would be valuable to add a normative perspective to the empirical investigation of particular issues. More broadly, the study indicates a need for more attention to ethical issues related to this normalized substance—including, but also beyond, addiction, reproductive harms, and individual freedoms versus the public good.

## Supplementary Information

Below is the link to the electronic supplementary material.

Supplementary file1 (DOCX 18 KB)

Supplementary file2 (DOCX 24 KB)

## Data Availability

The transcripts analysed for this article cannot be shared due to the possibility of re-identification of informants.

## Footnotes

1 Various terms were adopted by informants, with some using “dependence” or “addiction” with distinct meanings (e.g. using “dependence” to mean a biological state and “addiction” to mean a biopsychosocial state). As there was variation between informants I use the terms interchangably in what follows, and more specific terminology where appropriate.

2 The Stronger Futures legislation ceased after interviews were conducted, and cashless welfare cards have been phased out during 2022-23, though over 30,000 of people remain on income management of some form (DSS 2024). Laws against public drinking can be made at state/territory level or by local government, and include restricted zones and laws against public intoxication; reforms have been recently made in some areas (DHAC 2024).

3 Demand reduction includes treatment, and interventions in the social determinants of consumption. Harm reduction includes any measures that aim to reduce harm without necessarily reducing use, e.g. better late-night public transport; shatterproof glassware in venues.
