# Supplementary material for: Othering, Stigma, and Normalization: A Key Informant Interview Study on Ethical Issues in Alcohol Policy in Australia
Source: J Bioeth Inq. 2025 Aug 13;23(1):27–47. doi: 10.1007/s11673-025-10443-6 (PMC13068702; doi:10.1007/s11673-025-10443-6)
Supplement: Supplementary file 1 — Supplementary file1 (DOCX 18 KB) [file 11673_2025_10443_MOESM1_ESM.docx]

**Appendix 1: Interview guide**

**Conception and role of ethics**

- What does ‘ethics’ mean in your view?
- Do ethics or ethical discussion come up in your work (often/ever)? [If yes] How?
- Are there values that inform or motivate your research/policy work?

**Ethical issues in alcohol policy in Australia**

- What do you think are the most important ethical issues related to alcohol policy currently in Australia?
- Are there examples of practices or policies you think are ethically problematic? What is it about these that makes you regard them unethical?
- Do you think our drinking culture has ethical implications or aspects?
- We know that alcohol use is often heavily socially coded (norms differ by who is drinking, when, where, with whom, etc). Do you think these social codings of alcohol use have ethical implications in any sense?
