# Supplementary material for: Othering, Stigma, and Normalization: A Key Informant Interview Study on Ethical Issues in Alcohol Policy in Australia
Source: J Bioeth Inq. 2025 Aug 13;23(1):27–47. doi: 10.1007/s11673-025-10443-6 (PMC13068702; doi:10.1007/s11673-025-10443-6)
Supplement: Supplementary file 2 — Supplementary file2 (DOCX 24 KB) [file 11673_2025_10443_MOESM2_ESM.docx]

**Appendix 2: COREQ (COnsolidated criteria for REporting Qualitative research) checklist**

| Topic | Item No. | Guide Questions/Description | Answer | Page No. | |
| --- | --- | --- | --- | --- | --- |
| Domain 1: Research team and reflexivity | | | | | |
| Personal characteristics | | | | | |
| Interviewer/facilitator | 1 | Which author/s conducted the interview or focus group? | [blinded for review] | Title page/author details | |
| Credentials | 2 | What were the researcher’s credentials? Eg PhD, MD | PhD | Title page/author details | |
| Occupation | 3 | What was their occupation at the time of the study? | Lecturer in Philosophy | Title page/author details | |
| Gender | 4 | Was the researcher male or female? | Female | Title page/author details | |
| Experience and training | 5 | What experience or training did the researcher have? | Research track record in bioethics; experience in qualitative research; alcohol/drug policy experience | 4 | |
| Relationship with participants | | | | | |
| Relationship established | 6 | Was a relationship established prior to study commencement? | No | 5 | |
| Participant knowledge of  the interviewer | 7 | What did the participants know about the researcher? e.g. personal  goals, reasons for doing the research | Research aims, researcher’s research background | 4 | |
| Interviewer characteristics | 8 | What characteristics were reported about the interviewer/facilitator?  e.g. Bias, assumptions, reasons and interests in the research topic | Reasons for the study, interests in the research topic, relevant research and work experience | 4-5 | |
| Domain 2: Study design | | | | | |
| Theoretical framework | | | | | |
| Methodological orientation  and Theory | 9 | What methodological orientation was stated to underpin the study? e.g.  grounded theory, discourse analysis, ethnography, phenomenology,  content analysis | Reflexive thematic analysis (cf Braun and Clarke 2006, 2019, 2024) | 3-6 | |
| Participant selection | | | | | |
| Sampling | 10 | How were participants selected? e.g. purposive, convenience,  consecutive, snowball | Purposive | 3 | |
| Method of approach | 11 | How were participants approached? e.g. face-to-face, telephone, mail,  email | Email | 3 | |
| Sample size | 12 | How many participants were in the study? | 17 | 1, 4 | |
| Non-participation | 13 | How many people refused to participate or dropped out? Reasons? | 45 email invitations were declined (4) or not responded to (41). Declining invitees noted lack of time. No participants withdrew. | 3 | |
| Setting | | | | | |
| Setting of data collection | 14 | Where was the data collected? e.g. home, clinic, workplace | Zoom (as conducted during Covid lockdowns) / Interviewee’s location of choice | 4 | |
| Presence of non-  participants | 15 | Was anyone else present besides the participants and researchers? | No | 4 | |
| Description of sample | 16 | What are the important characteristics of the sample? e.g. demographic  data, date | Expertise in alcohol policy issues in Australia (key informant study) | 3 | |
| Data collection | | | | | |
| Interview guide | 17 | Were questions, prompts, guides provided by the authors? Was it pilot  tested? | Semi-structured nterview guide was used. No pilot testing. | | Appendix 1 and page 4 |
| Repeat interviews | 18 | Were repeat interviews carried out? If yes, how many? | No | | 4 |
| Audio/visual recording | 19 | Did the research use audio or visual recording to collect the data? | Yes (audio only was saved) | | 4 |
| Field notes | 20 | Were field notes made during and/or after the interview or focus group? | No | | NA - Field notes are not applicable to this type of study |
| Duration | 21 | What was the duration of the inter views or focus group? | 43-90 minutes | | 4 |
| Data saturation | 22 | Was data saturation discussed? | No. The method adopted following Braun and Clarke does not use the concept of saturation. For an argument agains appeals to saturation see Braun V, Clarke V. 2021. To saturate or not to saturate? Questioning data saturation as a useful concept for thematic analysis and sample-size rationales. *Qualitative Research in Sport, Exercise and Health*13 (2): 201-216. | | NA |
| Transcripts returned | 23 | Were transcripts returned to participants for comment and/or correction | No | | 4 |
| Domain 3: analysis and findings | | | | | |
| Data analysis | | | | | |
| Number of data coders | 24 | How many data coders coded the data? | 1 | Title page/author details | |
| Description of the coding tree | 25 | Did authors provide a description of the coding tree? | Yes | The coding tree is represented in table 2; description is on page 4 | |
| Derivation of themes | 26 | Were themes identified in advance or derived from the data? | Themes were identified in the data by the researcher. Note the method adopted (Braun and Clarke 2006, 2019) is developed in part out of the view that themes can never be purely ‘derived’ from the data, as they necessarily involve the researcher’s reflexivity. | 3-5 | |
| Software | 27 | What software, if applicable, was used to manage the data? | NVivo | 4 | |
| Participant checking | 28 | Did participants provide feedback on the findings? | No | 4 | |
| Reporting | | | | | |
| Quotations presented | 29 | Were participant quotations presented to illustrate the themes/findings?  Was each quotation identified? e.g. participant number | Yes | 5-20 | |
| Data and findings consistent | 30 | Was there consistency between the data presented and the findings? | Yes. The findings (themes/sub-themes/issues) were generated from analysis of the data (transcripts/recordings). Raw data cannot be made available without compromising participant anonymity. | 3-5 | |
| Clarity of major themes | 31 | Were major themes clearly presented in the findings? | Major themes are stated in the abstract, headings in the results section, and table 2. | 1, 5-20, 33-35 | |
| Clarity of minor themes | 32 | Is there a description of diverse cases or discussion of minor themes? | Minor themes are identified as sub-headings in the results section and in table 2. Diverse opinions are discussed where relevant throughout the results section. | 5-20, 33-35 | |
